# Salvage of Intraoral Dehiscence With a Superficial Temporal Artery Islandized Flap (STAIF)

**DOI:** 10.7759/cureus.58049

**Published:** 2024-04-11

**Authors:** Brian A Mailey, Blake M Sparkman, Alina K Sinha, Timothy Daugherty

**Affiliations:** 1 Department of Plastic Surgery, Southern Illinois University School of Medicine, Springfield, USA; 2 Plastic and Reconstructive Surgery, Saint Louis University School of Medicine, Saint Louis, USA; 3 Plastic and Reconstructive Surgery, University of Missouri Kansas City School of Medicine, Kansas City, USA; 4 Plastic and Reconstructive Surgery, Southern Illinois University School of Medicine, Carbondale, USA

**Keywords:** facial trauma, superficial temporal artery islandized flap, mandibular reconstruction, intraoral dehiscence, free fibula flap

## Abstract

Intraoral dehiscence compromises free fibula flaps following mandibular reconstruction. Salivary contamination risks thrombosis of microvascular anastomosis and hardware infection. The superficial temporal artery islandized flap (STAIF) provides a non-microsurgical reconstructive option for regaining intraoral competency for a time-sensitive complication. The STAIF is based on the superficial temporal artery coursing along the anterior hairline. The flap is mapped with the assistance of the Doppler probe. The width of the skin paddle is dependent upon the ability to close the donor site. The flap is taken down to the level of the zygomatic arch and tunneled into the mouth. We present a case of a patient who underwent mandibular reconstruction with a free fibula flap after a traumatic shotgun wound. The patient developed repeated intraoral dehiscence following failed local buccal and floor of mouth flaps leading to salivary contamination of the flap and hardware. The intraoral dehiscence was successfully salvaged on the third attempt with a STAIF. Intraoral dehiscence requires urgent attention to prevent loss of the free fibula flap after mandibular reconstruction. The STAIF is a non-microsurgical option for restoring intraoral competency. This robust, axially vascularized skin paddle may be split for intra- and extraoral coverage, as was performed in this case, and is an essential tool in the reconstructive armamentarium.

## Introduction

Intraoral dehiscence following mandibular reconstruction endangers free fibula flaps. Salivary contamination leads to anastomotic thrombosis, hardware infection, osteoradionecrosis, and flap loss [1]. The options for primary reconstruction to restore intraoral competence vary with defect size, location, tissue quality, and individual patient needs. The reported incidence of free fibula flap dehiscence ranges from 13% to 33% [1,2]. Secondary salvage options are limited and require time-sensitive interventions to prevent flap loss.

We present a case of repeat intraoral dehiscence following free fibular reconstruction of the mandible from a traumatic shotgun wound. After failed local buccal and floor-of-mouth flaps, the intraoral wound was successfully closed with the superficial temporal artery islandized flap (STAIF). The STAIF is an axially supplied flap consisting of superficial temporal fascia, subcutaneous tissue, and a versatile skin paddle centered over the vascular pedicle. Dependent upon the superficial temporal artery and vein as a vascular pedicle, the STAIF provides a richly vascularized tissue paddle, with broad functionality given its ease of harvest, rotational arc, size, and soft tissue flexibility.

## Case presentation

Case description

**Figure 1:**
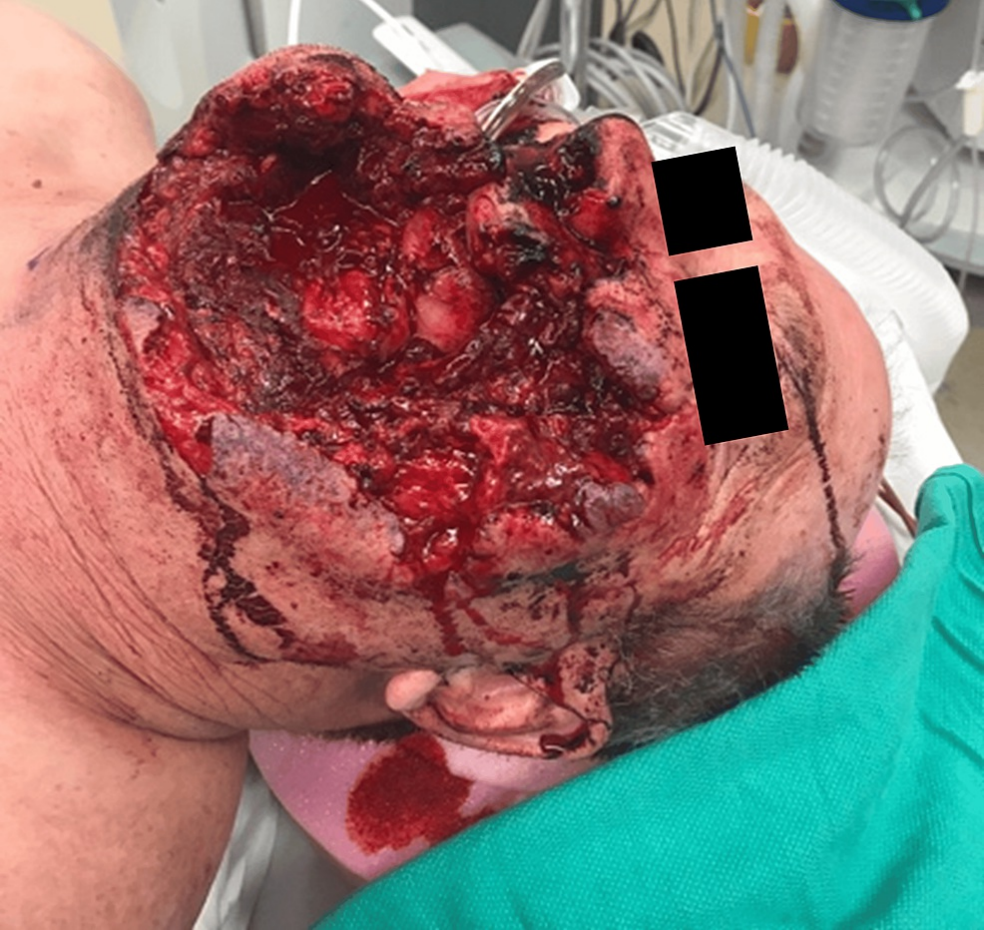
The patient on admission following a shotgun wound to the face during initial stabilization. He underwent tracheostomy and multiple debridements of devitalized tissue.

A 54-year-old male arrived at the regional trauma center following a self-inflicted gunshot wound to the face (Figure 1). 

**Figure 2:**
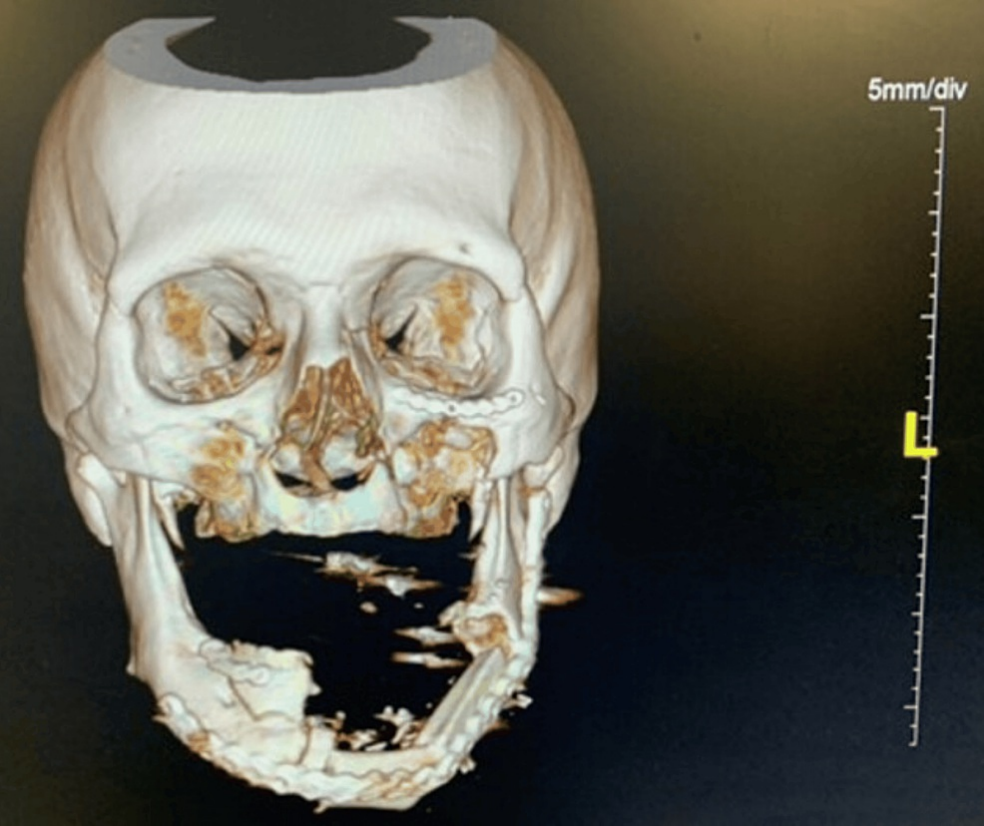
Three-dimensional modeling was used to create a cutting guide only. Mandibular and external soft tissue reconstruction was performed with an osteocutaneous free fibular flap.

The defect spanned the left side of the face inferolateral to the nose and inferior to the left eye. The defect had abraded and jagged edges. After stabilization, tracheostomy placement, and two rounds of debridement, a vascularized free fibula reconstruction was planned and performed using 3D modeling (Figure 2).

The intraoral defect was reconstructed using local tissues but dehisced following multiple attempts at intraoral closure. The initial closure attempts consisted of (1) local tissues at the time of initial flaps from the floor of the mouth and buccal lining tissue, followed by (2) spanning the defect with a thin piece of acellular dermal matrix (ADM) at a second operation, and (3) subsequently with another piece of ADM covered with a pedicled floor of mouth flap. These local flaps failed, exposing fibular bone and hardware. At the fourth operation, a split skin paddle STAIF was used for extra- and intra-oral coverage that led to stable coverage.

STAIF design

The design of the STAIF begins with identifying the superficial temporal artery (STA) via a Doppler probe. Typically, the STA is easily palpated approximately 2 cm anterior to the anterior helical rim and courses superiorly. In our experience, the flap may be elevated at least 5 cm past the last Doppler-able vessel location. After identifying the vascular course, the skin is pinched to visualize how much can be closed primarily. Marks are made to accommodate the primary closure of the donor site.

STAIF dissection

**Figure 3:**
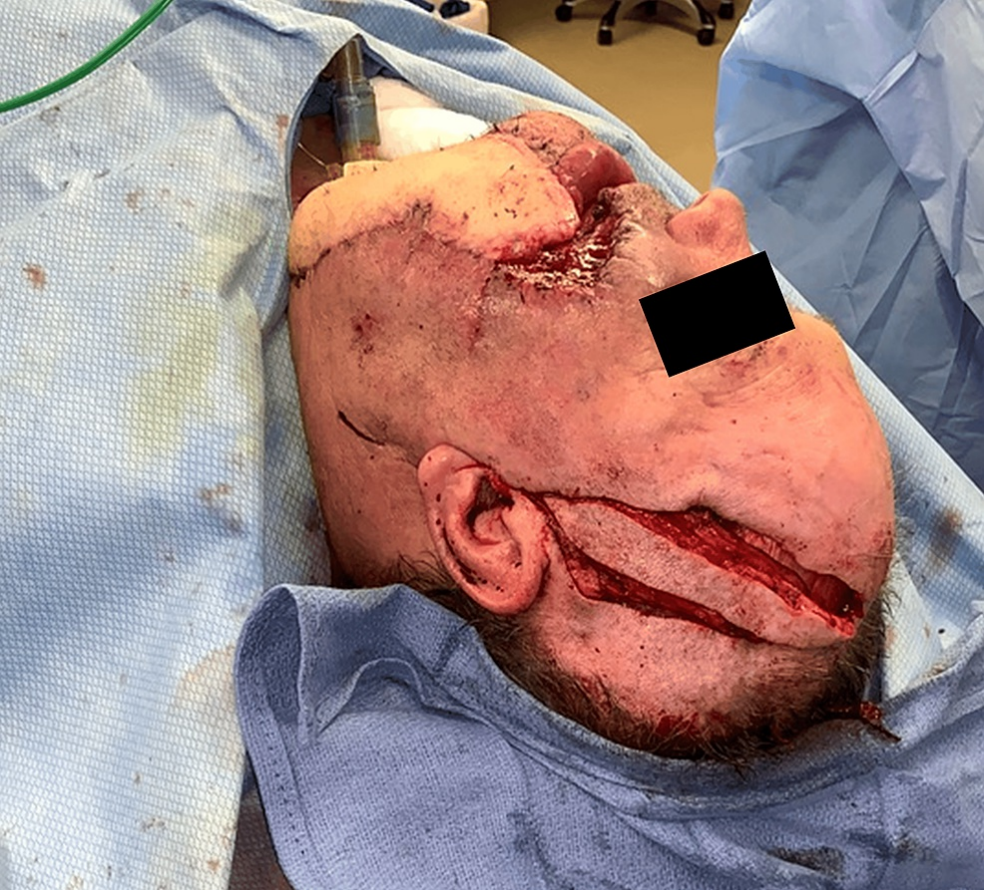
Harvest of the superficial temporal islandized artery flap (STAIF) pedicle following Doppler probe usage for mapping.

The skin paddle is incised and elevated in a superior to inferior direction. Additional temporoparietal (TP) fascia, galea, or pericranium may be included via undermining for more extensive coverage or fascial wrapping of the fibula or hardware. In this case, the TP, STA, and fascia were dissected down to the level of the temporalis muscle, keeping the pedicle with the flap. Dissection was maintained posterior to the course of the frontotemporal branch of the facial nerve, which lies immediately deep to the TP fascia. The skin paddle was islandized, and the flap elevated to the level of the zygomatic arch. A tunnel was made in the cheek superficial to the zygomatic arch at the level of the tragus. The flap was passed into the mouth, crossed the midline of the mandibular gingivobuccal sulcus, and sutured intraorally to cover the gingivobuccal sulcus of the lower lip and mucosal defect in the floor of the mouth (Figure 3).

For more extended reach, the flap may be passed under the zygomatic arch. In this case, the skin paddle was divided and split, providing both intra- and extra-oral coverage, like the methods described by Elbanoby et al. [3]. The distal skin paddle served for intraoral defect coverage; the proximal allowed for the reconstruction of the cheek and outer mouth after partial de-epithelization. The external facial defect was in the beard line and reconstructed with hair-bearing skin. The donor site was closed primarily after wide undermining and healed favorably. A Doppler signal was identifiable in each skin paddle.

Patient outcome

**Figure 4:**
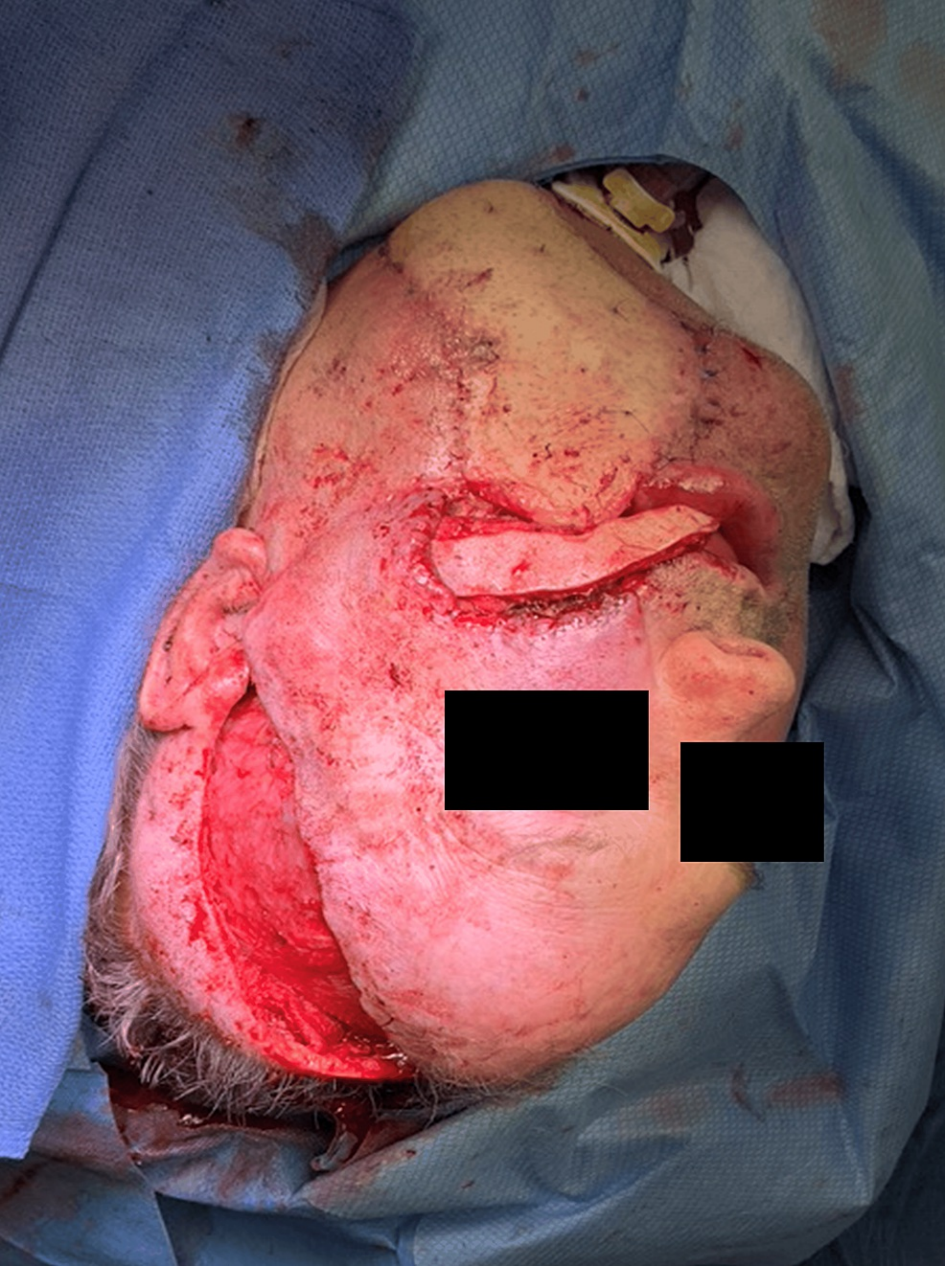
Dissection carried through temporoparietal fascia for tunneling anterior to tragus and tunneling through a zygomatic arch to intraoral defect complete.

Intraoral competence was restored following the STAIF use in this patient. Coverage of the fibula and hardware was obtained without further dehiscence or exposure (Figure 4).

**Figure 5:**
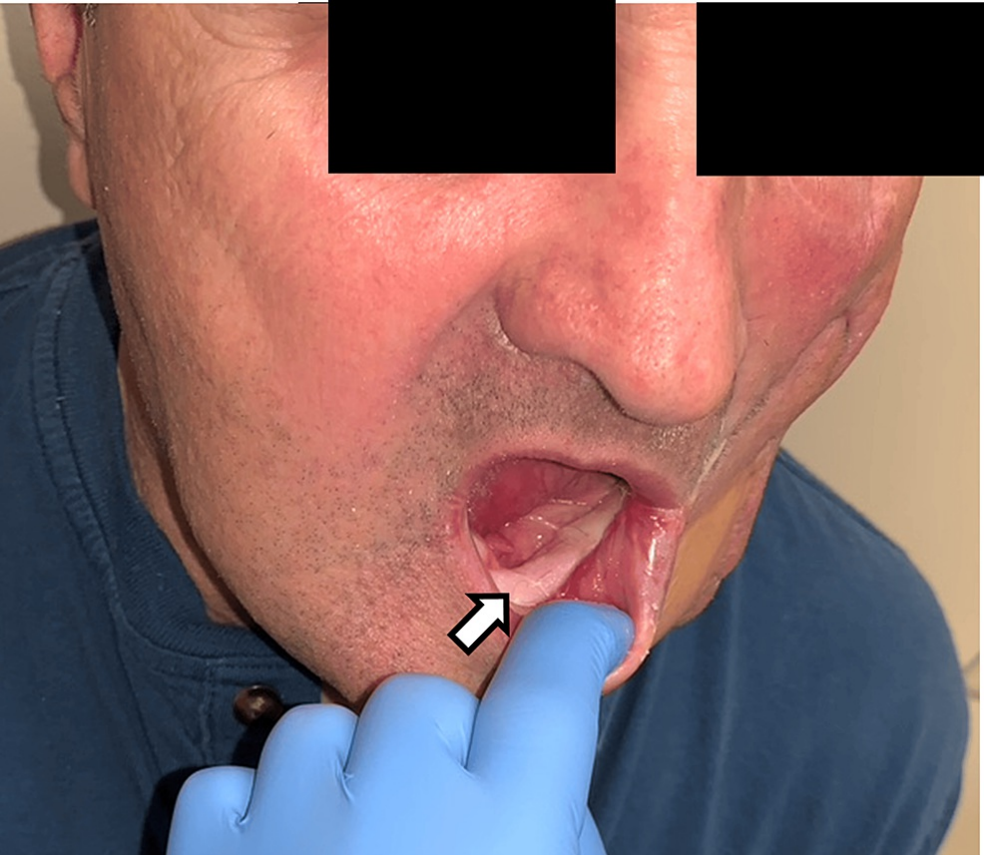
Demonstration of restored intraoral competence following the superficial temporal islandized artery flap (STAIF) use after failed reconstructive attempts including local advancement, the floor of the mouth, and acellular dermal.

**Figure 6:**
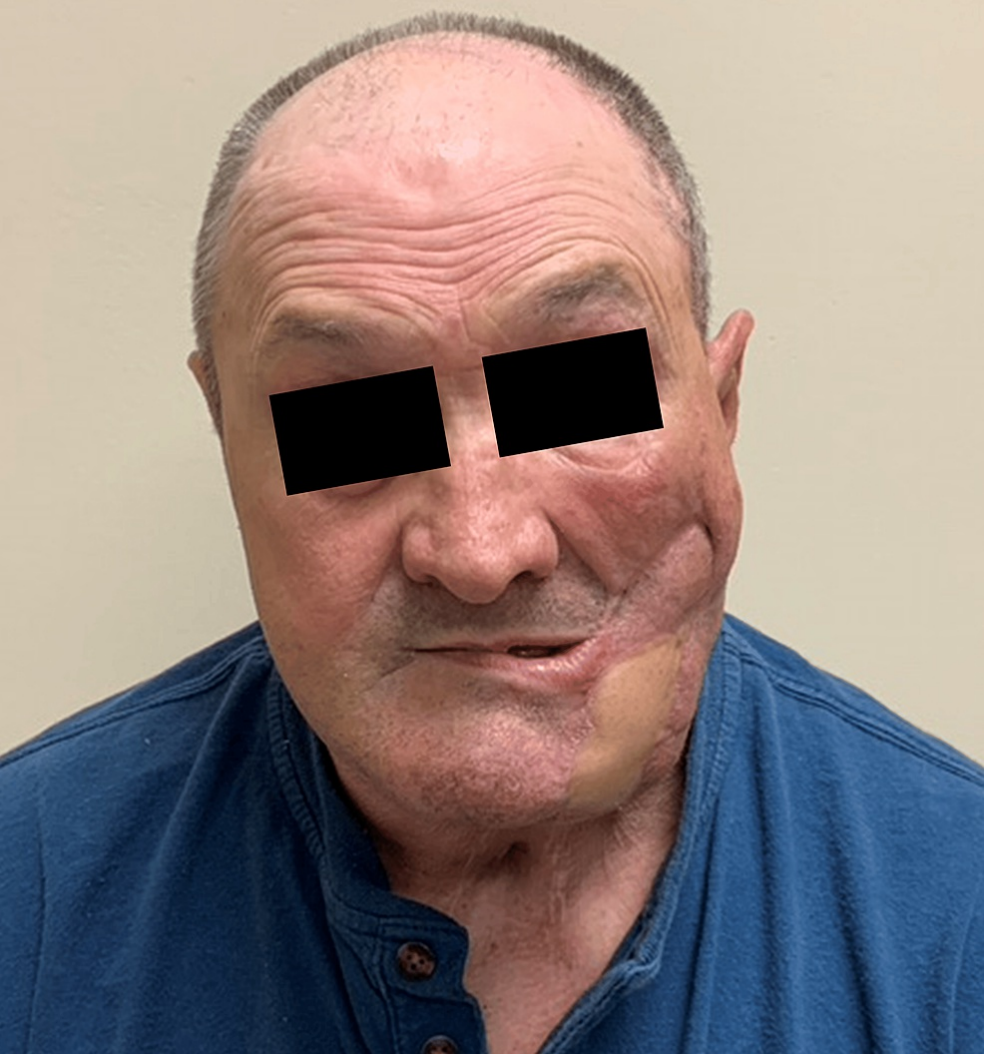
Anterior view of cosmetic outcome following the superficial temporal islandized artery flap (STAIF) use.

He returned two months postoperatively for debulking and flap inset given an element of microstomia and difficulty with lip elevation. The flap was further divided at its tip into two portions for improved intra- and extraoral delineation and enhanced lip commissure definition. The split skin paddle STAIF successfully reconstructed intraoral defects and external cheek soft tissue wounds (Figures 5, 6).

## Discussion

The concept of a flap based on the superficial temporal artery was introduced in 1966 by McGregor and Reid as a temporal flap based on the anterior branch of the superficial temporal vessels [4]. In 1974, Bakamjian carried the concept of using superficial temporal vessels in a staged approach for intraoral reconstruction [5]. A variety of island flaps can be based on the STA with variable tissue composition. The STAIF can be an osteocutaneous flap by taking the outer cortex of the calvarial bone. Additionally, it may be harvested with or without a skin paddle. Furthermore, it may be split into multiple paddles as described by Elbanoby et al. and used for both intra- and extraoral reconstruction as a salvage option for free flap coverage, as described in this patient case [3]. 

Flap planning is accomplished via digital palpation, Doppler localization techniques, indocyanine green laser angiography, and thermal imaging [6]. The STAIF’s consistent ease of harvest and rotational arc allows for local facial reconstruction or intraoral tunneling for cavity resurfacing. Cavity resurfacing with STAIF is applicable for defect coverage from many etiologies, including oncologic, trauma, burns, exposed hardware, and congenital cases [3,7]. Useful for intraoral reconstruction and facial defect coverage, simultaneous reconstructive needs may be met with dividing and sliding techniques [8]. The desired flap length is dissected to the level anterior to the zygomatic arch, and a subcutaneous tunnel is created. Though some reports suggest tunneling may cause venous congestion, our experience and reports from other groups indicate tunneling is safe with minimal risk for flap compromise [6,7,9]. The STAIF donor site can be closed primarily or skin grafted if a more prominent skin paddle is needed. Donor site morbidity could include widened scars or alopecia (Figure 3B and Figure 4A).

Herein, we describe the first use of a split skin-paddle STAIF for intra- and extraoral coverage for successful free flap salvage from intra-oral contamination. The patient required an additional thinning, debulking, and flap-splitting procedure to achieve a more optimal outcome. This step is routinely required to achieve adequate mouth opening and reduce tethering to the cheek but is a short, outpatient procedure. The STAIF can also be used in a primary setting to circumvent the need for multiple free flaps. We now reconsider it as a primary option for intraoral reconstruction.

## Conclusions

Addressing intraoral dehiscence in the setting of mandibular reconstruction demands timely consideration given salivary contamination and concern for anastomotic thrombosis, infection, and flap loss. The first-line choice for intraoral dehiscence following a free fibula flap has traditionally relied on free tissue transfers, such as free radial forearm flaps and anterolateral thigh perforator flaps. These microvascular flaps are challenging in the subacute postoperative dehiscence period; further, they are often bulky. Local tissue options for secondary reconstruction are limited. The role of biomaterials, such as acellular dermal matrix for intraoral closure, may be used to temporize a defect; however, their tensile strength and reliability in intraoral dehiscence have yet to be established. The STAIF offers a well-vascularized, convenient, flexible, and reliable non-microsurgical option for intraoral dehiscence with minimal donor site morbidity. STAIF continues to be a vital tool in the reconstructive armamentarium for the reestablishment of intraoral competency.
